# Holm Oak (*Quercus ilex*) Transcriptome. *De novo* Sequencing and Assembly Analysis

**DOI:** 10.3389/fmolb.2017.00070

**Published:** 2017-10-06

**Authors:** Victor M. Guerrero-Sanchez, Ana M. Maldonado-Alconada, Francisco Amil-Ruiz, Jesús V. Jorrin-Novo

**Affiliations:** ^1^Agroforestry and Plant Biochemistry, Proteomics and Systems Biology, Department Biochemistry and Molecular Biology, Universidad de Córdoba, Cordoba, Spain; ^2^Servicio Central de Apoyo a la Investigación, Universidad de Córdoba, Cordoba, Spain

**Keywords:** Holm oak, *Quercus ilex*, RNA-sequencing, assemblers, illumina

## Introduction

Holm oak (*Quercus ilex* L. subsp. *ballota* [Desf.] Samp.) is the dominant tree species in the Mediterranean forest with great ecological and economic value (Pulido et al., 2001). It constitutes, together with cork oak (*Q. suber)*, the “dehesa,” a typical Mediterranean agro-forestry-pastoral ecosystem, covering almost four million hectares in the western Iberian Península (Joffre et al., 1999). Besides, holm oak is widely used in reforestation programs and silvicultural practices, being their seeds, acorns, used for feed, and fatten the exclusive Iberian race pigs, whose meat is the basis of a high-quality food industry (Vicente and Alés, 2006; Cañellas et al., 2007).

Nowadays, *Q. ilex* forest maintenance and sustainability are facing severe problems and challenges. Those are related to agricultural practices, low natural regeneration, seed viability, which may be due to their non-orthodox seed character (Doody and O'Reilly, 2008), plant mortality in both adult trees and young plants after field transplantation resulting from adverse environmental conditions like drought, the so-called decline syndrome (Gallego et al., 1999), especially considering the current and future climate change scenario (Plieninger et al., 2004; Bates et al., 2008; Corcobado et al., 2013). Overcoming those threats could be greatly facilitated if olm oak ecophysiological behavior was better understood at the molecular level. Nowadays, multidisciplinary approaches by integrating the so-called—omic studies—transcriptomics, proteomics and metabolomics—have become indispensable to shed light on the fine-tuned molecular regulation in many biological systems/species. Thus, system biology aims to describe and interpret the full complexity of cells, tissues, organs, and organisms.

In this context, our research group has been investigating different aspects of *Q. ilex* biology such as natural variation, seed germination and seedling growth, physiology, biotic and abiotic stress-responses, combining classical biochemistry, and integrating those multidisciplinary “omics” analysis (Echevarría-Zomeño et al., 2009, 2012; Jorrín-Novo et al., 2009; Valero-Galván et al., 2011, 2012, 2013; Sghaier-Hammami et al., 2013, 2016; Romero-Rodríguez et al., 2014). Nevertheless, the scarce genomic information (to date) available for *Q. ilex*, supposes, such as for other orphan tree species (Abril et al., 2011; Jorrín-Novo et al., 2015), a notable obstacle to successfully carry out these global studies at molecular level. Driven by that need, our main aim has been to generate a reference transcriptome of *Q. ilex* which will support and complement future research within this species. For that purpose, as a first approach we sequenced the mRNA of a pooled plant sample containing equal amounts of homogenized tissue from acorn embryo, leaves, and roots, using an Illumina Hiseq 2500 platform. Contrasting different assembly strategies and algorithms, we present here the first *de novo* assembled transcriptome of the non-conventional plant *Q. ilex*.

The pre-processed raw reads generated by the sequencing platform, and used for the *de novo* assembly, have been deposited at the NCBI SRA database with accession number SRR5815058.

This new genomic resource will set the stage for ongoing and future studies to obtain a better understanding of molecular mechanisms involved in physiological processes such as seed germination, seedling establishment, drought, which are essential for selection of superior phenotypes or Candidate Plus for restoration and reforestation programs under the impending climate change in Mediterranean regions.

## Materials and methods

### Plant material

Mature acorns from Holm oak (*Q. ilex* L. subsp. *ballota* [Desf.] Samp.) were collected from a tree located in Aldea de Cuenca (province of Córdoba, Andalusia, Spain). Acorns were germinated and seedlings grew in a chamber under controlled conditions (a 12 h photoperiod, a temperature of 21 ± 1°C, a relative humidity of 60 ± 5% and an irradiance of 200 μmol m^−2^ s^−1^, Echevarría-Zomeño et al., 2009). Germinated embryo, leaves and roots from 1 year plantlets were collected separately, weighted, and individually frozen in liquid nitrogen. The plant material used for RNA sequencing experiments consisted in a pool generated by mixing equal amounts of homogenized tissue from acorn embryo, leaves, and roots.

### RNA extraction

Total RNA was extracted from 50 mg pooled plant sample according the procedures previously set up in our laboratory for *Q. ilex* samples (Echevarría-Zomeño et al., 2012). Contaminating genomic DNA was removed by DNase I (Ambion) treatment. Total RNA was quantified spectrophotometrically (DU 228800 Spectrophotometer, Beckman Coulter, TrayCell Hellma GmbH & Co. KG). The high quality and integrity of the RNA preparation was tested electrophoretically (Agilent 2100 Bioanalyzer). Only high-quality RNAs with RIN values > 8 and A_260_:A_280_ ratios near 2.0 were used for subsequent experiments.

### Enrichment of mRNA, cDNA synthesis, and library generation for illumina HiSeq 2500 platform. paired-end sequencing

The library construction of cDNA molecules was carried out using Illumina TruSeq Stranded mRNA Library Preparation Kit according to manufacturer instructions using 2 μg of total RNA followed by poly-A mRNA enrichment using streptavidin coated magnetic beads and thermal mRNA fragmentation. The cDNA was synthesized, followed by a chemical fragmentation (DNA library) and sequenced in the Illumina Hiseq 2500 platform, using 100 bp paired-end sequencing (Conesa and Götz, 2008; De Wit et al., 2012).

### *De novo* assembly and analysis of high throughput RNA sequencing data

The raw reads obtained from the sequencing platform were pre-processed in order to retain only high-quality sequences to be subsequently used in the assembly. Thus, each original sequence was quality trimmed considering several parameters (quality trimming based on minimum quality scores, ambiguity trimming to trim off e.g., stretches of Ns, base trim to remove specified number of bases at either 3′ or 5′ end of the reads). The pre-processing parameters used were selected as following: trimming sequences by maximum 2 ambiguous nucleotides), minimum mean quality assuming error probability < 0.01, and filtering out those sequences shorter than 30 nucleotides.

**Figure 1:**
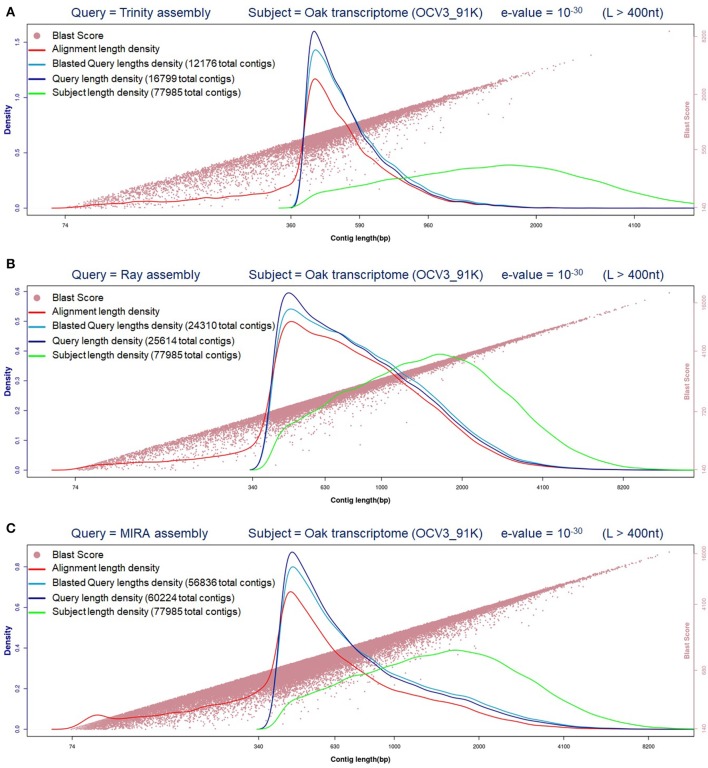
Evaluation of *Q.ilex* transcriptomes generated. Contig (longer than 400 nucleotides = L > 400 nt) length distribution and comparative evaluation against oak transcriptome (BlastN e-value = 10^−30^). **(A)** Trinity; **(B)** Ray; **(C)** MIRA.

Three different assemblers were employed to *de novo* assemble the *Q. ilex* transcriptome, considering there is not a reference genome available, and further evaluated to contrast the results obtained (Figure 1).

Trinity 2.4.0. performs a *de novo* assembly using an algorithm based on Bruijn graphs (Grabherr et al., 2011). For the assembly, Trinity 2.4.0 was launched with a k-mer value (*k* = 25).

Ray 2.3.1. assembly uses de Bruijn graphs but its framework is not based on the Eulerian steps. Specific subsequences, seeds, are defined, and for each of them, the algorithm extends it to a contig. Heuristics are defined that control the extension process in such a way that the process stops if, at some point, the readings family does not clearly indicate the address of the extension (Boisvert et al., 2010). In this case we selected a k-mer value of 31.

MIRA 4.9.6 software (Chevreux et al., 1999), unlike Trinity and Ray, is based on the strategy known as Overlap /Layout/ Consensus. Following the author guidelines/recommendations for Illumina data, we used the complete raw data without a filtering process like we described previously.

Evaluation of the structure of the generated assemblies was done with the QUAST software (Gurevich et al., 2013).

The assemblies obtained using the three aforementioned softwares were blasted (e-value of 10^−30^) against the most accurate and nearest phylogenetic transcriptome currently available, the oak transcriptome (containing *Quercus robur* and *Quercus petraea* sequences) (Lesur et al., 2015). That transcriptome database is divided in two files OCV3_91K and OCV3_101K but OCV3_91K has a larger amount of valuable information of *Quercus* spp. transcriptome. So, we chose OCV3_91K as a general oak transcriptome database.

## Results

### Evaluation and annotation of the assembled transcriptomes

There are differences between the three assembled transcriptomes in terms of transcriptome architecture/structure. Thus, the N50 value, number of contigs and the average length of the sequences generated by each algorithm differ (Table 1).

**Table 1:** Assembly structure and similarity with oak transcriptome.

**Number of original raw reads**	**55275472**		
	**MIRA**	**Ray**	**Trinity**
# contigs (≥0 bp)	169449	107487	77159
# contigs(≥500 bp)	43014	20495	8803
# contigs (≥1,000 bp)	15445	8773	696
# contigs (≥5,000 bp)	155	73	1
# contigs (≥10,000 bp)	2	3	0
Largest contig	11254	12220	5916
Total length (≥0 bp)	83639406	41292773	26286544
Total length (≥1,000 bp)	27409911	14778197	904440
Total length (≥5,000 bp)	941227	471829	5916
Total length (≥10,000 bp)	21731	34168	0
N50	1211	1260	661
N75	742	827	563
L50	11473	5863	3428
L75	23813	11529	5931
GC (%)	41.69	42.47	39.14
Oak transcripts^*^ present in Q. ilex^**^	73073	63950	49679
Oak transcripts^*^ absent in Q. ilex^**^	13943	23066	37337
% of oak^*^ transcripts in Q. ilex^**^	83,98	73,49	57,09

*Comparison of Q. ilex transcriptome assembly using Trinity, RAY, and MIRA assemblers. Statistics and structure of the transcriptome assembly are indicated, including the number of contigs obtained of a minimum length (QUAST output data). Comparative hits with oak transcriptome are shown indicating the number of genes shared with oak and those newly found in Q. ilex*.

* Oak total transcripts = 87016;

** *BlastN with e-value = 10^−30^*.

Considering these results, we can state that MIRA generated more and longer contigs than RAY and Trinity (MIRA>RAY>Trinity), suggesting that a more robust architecture/structure is obtained by MIRA for the *Q. ilex* transcriptome assemby. Upon the continuous development of NGS methods, data processing, and transcript assemby remains a main challenge. Several studies have been published devoted to evaluate different *de novo* assemblers varying in performance and quality in terms of number and length of transcripts and computational speed (Clarke et al., 2013). Besides, it has been reported that the quality of the assembly using a given software depends on the biological sample on study (Bradnam et al., 2013). Thus, these aspects should be taken into consideration when comparing different softwares.

The comparison between the sequences generated from *Q. ilex* and those available from the close species, oak transcriptome, reveals that MIRA assembly was the one which shared the higher number of transcripts (73073), followed by RAY assembler (Table 1). Besides, MIRA assembly sequences blasted against oak transcriptome render the longest alignment lengths and better blast scores (Figure 1).

Taking into consideration the data and parameters evaluated (Table 1 and Figure 1), we decided to use the MIRA assembly to continue with the corresponding annotation of *Q. ilex* transcriptome. After blastX was completed against Uni-Prot (Swiss-Prot) curated database (e-value of 10^−5^), followed by the corresponding mapping process, 31973 annotated sequences were obtained by Blast2GO (Conesa and Götz, 2008).

## Direct link to deposited data

The pre-processed raw reads of the transcriptome assembly generated by the sequencing platform, and used for the de-novo assembly, have been deposited at the NCBI SRA database with the following accession number SRX2993508 and direct link: ftp://ftp-trace.ncbi.nih.gov/sra/sra-instant/reads/ByRun/sra/SRR/SRR581/SRR5815058/SRR5815058.sra

## Author contributions

AM: Collected samples, performed RNA isolation. VG and FA: Bioinformatic analysis of the data. VG, FA, JJ, and AM: Wrote the manuscript. JJ: Supervised the Project and acquired funding.

### Conflict of interest statement

The authors declare that the research was conducted in the absence of any commercial or financial relationships that could be construed as a potential conflict of interest.
